# Esthetic Rehabilitation with No-Preparation Veneers Applying BOPT: A Case Report with a 12-Month Follow-Up

**DOI:** 10.1155/2024/6620612

**Published:** 2024-06-28

**Authors:** Dima Alawa, Mawia Karkoutly, Hussam Milly

**Affiliations:** ^1^ Department of Cosmetic Dentistry Dental Institute Damascus University, Damascus, Syria; ^2^ Department of Pediatric Dentistry Damascus University, Damascus, Syria; ^3^ Department of Conservative Dentistry and Endodontics Dental Institute Damascus University, Damascus, Syria

## Abstract

A 45-year-old female patient was referred to the Department of Cosmetic Dentistry, Damascus University, seeking to improve dental aesthetics. The clinical examination showed a low smile line and microdontia in the maxillary arch. The treatment plan consisted of applying no-prep veneers with gingival modification, which is described in the biologically oriented preparation technique (BOPT) as a gingitage technique. The gingitage of the sulcus was performed using a yellow ring diamond flame with an inclination of 45 degrees, which causes it to bleed and creates a space between the internal wall of the sulcus and the axial wall of the tooth. After a 12-month follow-up, the gingiva was free of inflammation, recession, and plaque, and there was no increase in probing depth, according to the modified gingival index (MGI), gingival recession index (GRI), Silness-Loe plaque index (PI), and the community periodontal index of treatment needs (CPITN), respectively. The porcelain veneers were intact, with no chippings, cracks, and marginal discoloration. The patient was satisfied with her new smile.

## 1. Introduction

A smile is the most powerful communication approach between humans, and an attractive smile is a smile that looks natural and healthy. Conservative cosmetic treatment options are more favorable, which contributed to the minimally invasive dentistry approach [1]. Recently, the new biomimetic ceramic materials required reduced thickness, which led to a noticeable reduction of preparation invasiveness and optimal preservation of tooth structure. No-prep veneers are the best treatment option due to the maximum preservation of tooth structure [1, 2]. However, they have several limitations, including esthetic and periodontal complications because of overcontoured restoration that could alter the emergence profile [1–3]. The apical migration of the gingival margins is the main shortcoming of fixed prosthodontics, which compromises the esthetic aspects [4]. The biologically oriented preparation technique (BOPT) was proposed as an alternative, which results in optimal symmetry and better management of periodontal tissue when applying ceramic veneers. In addition, it offers multiple biological advantages, such as repositioning the emergence profiles and increasing the thickness of the gingiva and the gingival margin stability [5, 6]. The aim of induced bleeding BOPT is to facilitate the access of immature connective tissue cells and epithelial stem cells and stimulate cell differentiation, which leads to the formation of new periodontal tissue [7]. This case report presents the case of a patient with multiple diastemas who was treated with no-prep veneers with the gingitage technique.

## 2. Case Presentation

**Figure 1:**
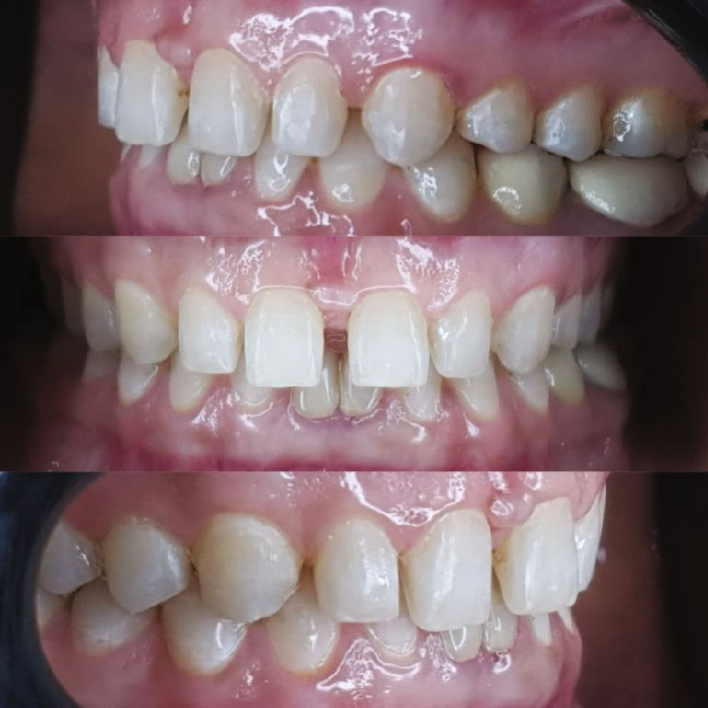
Initial intraoral photographs.

**Figure 2:**
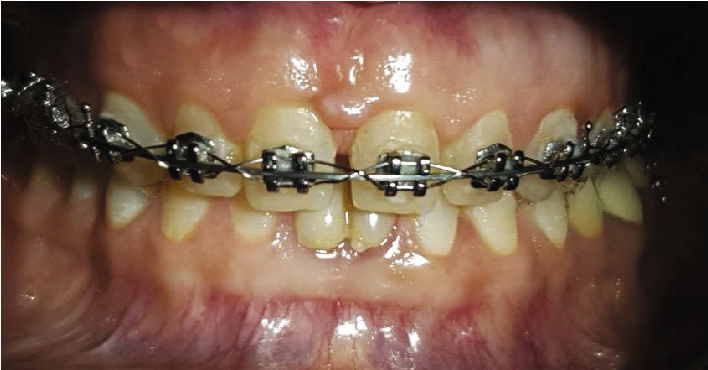
Orthodontic preparation.

**Figure 3:**
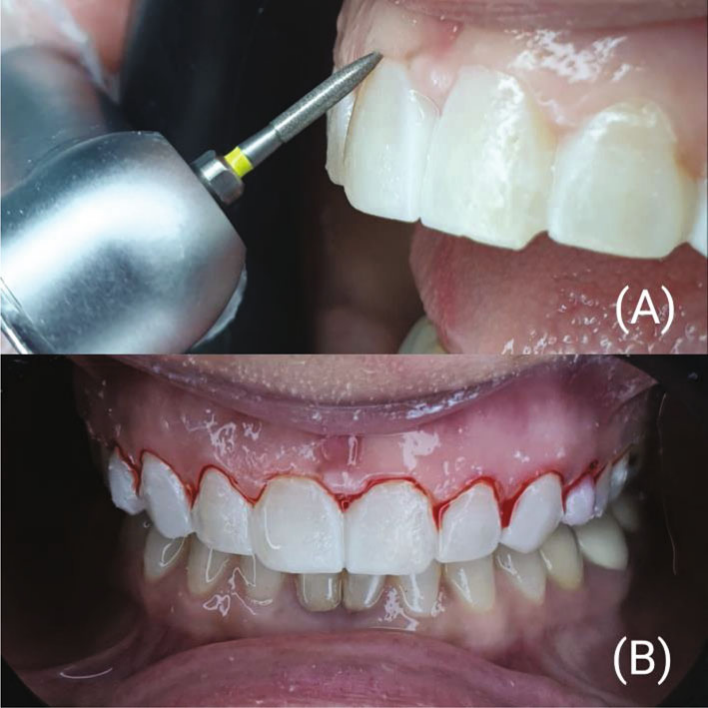
(A) Gingitage of the sulcus using fine-(50 *μ*m) grain flame bur placed on the most apical point of the temporary restoration with an angle of approximately 45° with tooth axis. (B) Gingitage of the sulcus after the application of mock-up.

A 45-year-old female patient was referred to the Department of Cosmetic Dentistry, Damascus University, seeking to improve dental aesthetics. In diagnostic assessment, extraoral examination showed no swelling or facial asymmetry. The intraoral examination showed a low smile line and microdontia in the maxillary arch. No dental caries, enamel defects, or parafunctional habits were detected [8]. The gingiva was free of inflammation, recession, and plaque, according to the modified gingival index (MGI) [9], gingival recession index (GRI) [10], and Silness-Loe plaque index (PI) [11], respectively. In addition, the periodontal status was assessed using the community periodontal index of treatment needs (CPITN) [12] (Figure 1). The treatment plan consisted of applying no-prep veneers with gingival modification, which is described in BOPT as a gingitage technique. Veneers were indicated to manage the residual space of the microdont teeth [8]. Orthodontic preparation was performed to distribute the dental spaces according to the golden ratio (Figure 2). The orthodontic preparation was performed using the continuous arch technique with MBT brackets 0.022. The treatment duration was 9 months, and then a vacuum fixation device was made [13]. At the first visit, intraoral and extraoral photographs were obtained to analyze the cosmetic aspects. During the second session, an accurate intrasulcular mapping is performed with a periodontal probe (WHO Probe 550B, LM-Dental™, Pargas, Finland) to assess the level of the epithelial attachment by measuring the distance from the cementoenamel junction to the periodontal pocket depth [14]. The pocket depth was measured according to CPTIN [12]. A gingival retraction was performed before dental impressions using an epinephrine-impregnated cord (Z-Twist, Gingi-Pak®, California, USA); then, the dental impression was made to build the diagnostic wax-up with the addition silicone (Soft Putty, B&E Korea Co., Ltd., Gyeonggi-do, Korea), and the bite registration was performed using a dental wax sheet (Polywax, Bilkim Ltd., Co., Izmir, Turkey). A cosmetic mock-up was performed to provide the patient with a three-dimensional view of her new smile [15]. First, a silicone matrix was made by molding the diagnostic wax-up with condensation silicon (Zetaplus, Zhermack, Badia Polesine, Italy). Second, spot etching with phosphoric acid etching gel 37% (Condac 37, FGM, São Paulo, Brazil) and bonding (Ambar, FGM, São Paulo, Brazil) were done on the maxillary teeth. This matrix was filled with a bis-acryl temporary resin material (Trantemp Crown, Nexobio, Chungcheongbuk-do, Korea), which was positioned over the teeth for approximately 1 minute. After the silicon matrix was removed, the mock-up remained mechanically attached to the teeth [16]. Once the mock-up was put, the gingitage of the sulcus was performed using fine-grain flame bur 30 *μ*m) (Dentsply, Maillefer, Ballaigues, Switzerland) placed on the most apical point of the temporary restoration and moved mesially and distally with irrigation and an angle of approximately 45° with tooth axis to maintain a convex profile, which causes it to bleed and creates a space between the internal wall of the sulcus and the axial wall of the tooth (Figure 3). This space will fill with blood, which will later form a clot [17].

**Figure 4:**
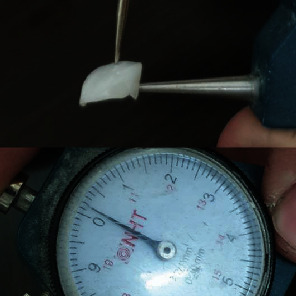
Thickness of porcelain veneer.

**Figure 5:**
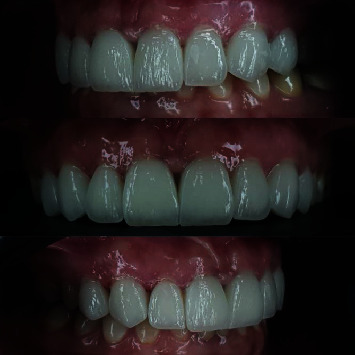
Intraoral view of veneers directly after cementation.

**Figure 6:**
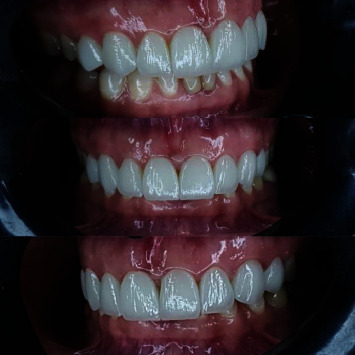
Intraoral view of veneers at a 9-month follow-up visit.

**Figure 7:**
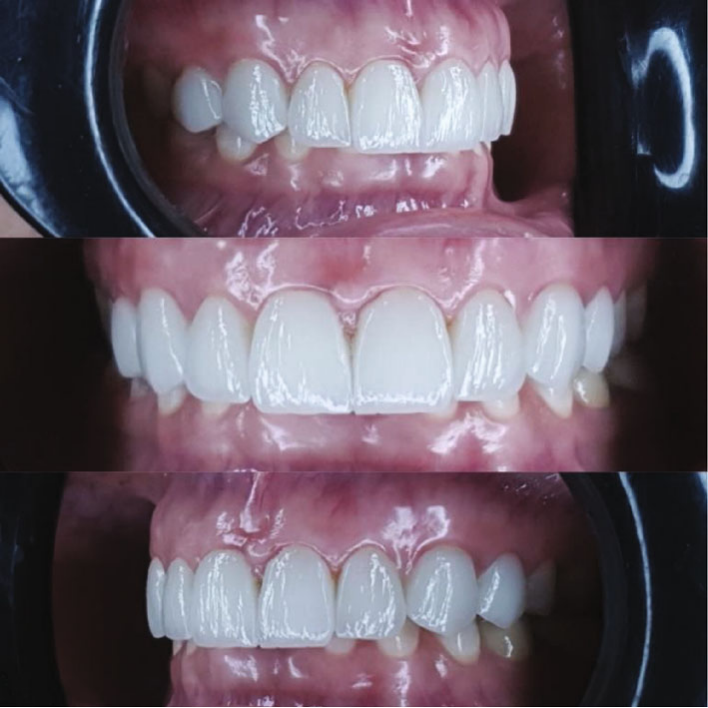
Intraoral view of veneers at a 12-month follow-up visit.

**Figure 8:**
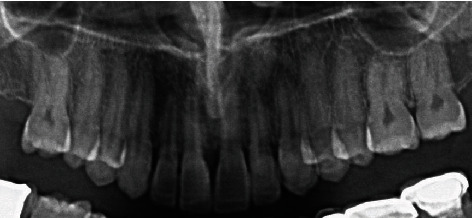
Radiographic photograph of veneers at a 12-month follow-up visit.

The mock-up was placed for 4 weeks and then removed to apply the try-in for the no-prep veneers [6]. All restorations were constructed with lithium disilicate (Figure 4). The gingival adaptation and the dental fit of the restorations were evaluated before cementation; then, a milky bright color was selected for cementation (Choice™ 2, Bisco, Illinois, USA). The treatment of the restoration surface before cementation was performed. Etch with 9.5% hydrofluoric acid gel (Porcelain etchant, Bisco, Illinois, USA) for 20 seconds and then thoroughly wash with water and dry for 1 minute. Apply the silane agent (Bis-Silane, Bisco, Illinois, USA) and then wait 1 minute for the silane agent to evaporate. Apply the adhesive cement (Tetric N-Bond Universal, Ivoclar Vivadent, New York, USA) on ceramic veneers, and cover it to prevent polymerization. At the same time, the treatment of the dental surface and cementation were done. Clean with a polishing brush (Pro-Brush™, Kerr, California, USA) and a stream of water, then isolate teeth with cotton rolls, etch with phosphoric acid-etching gel 37% (Condac 37, FGM, São Paulo, Brazil) for 30 seconds, and then wash and dry. Apply the adhesive without light activation. Position all ceramic veneers starting with the central teeth, primary light activation for 5 seconds, then removes excess cement with a dental probe (Dental Probe 23 Explorer, Medentra Plus, New Jersey, USA) and dental floss (Essential Floss, Oral-B Laboratories, Inc., Iowa, United States), final light activation for 40 seconds, and then adjust the occlusion with articulating paper (Articulating Paper, Osakadent, Guangdong, China) (Figure 5) [18]. Finally, give the patient instructions for maintaining oral hygiene. The patient was instructed to brush and floss regularly. To avoid scratching the veneers, the patient was instructed to use a nonabrasive toothpaste with a soft-bristled toothbrush. In addition, alcohol-containing mouthwashes were not recommended, and the patient was instructed to use alcohol-free mouthwashes [19]. The patient was recalled to the cosmetic clinic at Damascus University after one week, 3, 6, 9 (Figure 6), and 12 months (Figures 7 and 8) to check the health of soft tissues and to observe changes in the gingiva and veneers [20]. During the follow-up sessions, it was noted that the gingiva was free of inflammation, recession, and plaque, and there was no increase in probing depth from the baseline. The porcelain veneers were intact, with no chippings, cracks, and marginal discoloration. The patient was completely satisfied with the absence of need for preparation, anesthesia, and the absence of pain during and after the treatment, as well as the high aesthetics of the porcelain veneers that she obtained. The checklist of the procedures described in the present report is presented in Table 1.

## 3. Discussion

The main problem of not having a finish line for no-prep veneers is the excessive convexity of the tooth, which will give a bulky appearance and make it difficult to achieve the emergence profile, which will cause gingival irritation and periodontal problems [1]. Therefore, after case selection and periodontal examination, we opted to apply no-prep veneers with BOPT, which would make it possible to achieve gingival stability in the short and long terms and improve the restoration emergence profiles. Furthermore, this approach offers several biological advantages, such as repositioning the emergence profiles, correcting the cementoenamel junction (CEJ), increasing the thickness of the gingiva and the gingival margin stability [6], controlling the gingival groove invasion, and positioning the prosthetic finishing line inside the groove without invading marginal adaptation [7, 12]. The chief complaint was cosmetic discomfort with the diastema, and the case could be treated without dental preparation. Thus, it was possible to eliminate the problems of traditional porcelain veneer preparation that requires local anesthesia and the removal of enamel to achieve a thickness of at least 0.5-0.7 mm for the veneer [1, 21, 22]. The treatment was started with dental impressions; then, a mock-up was accomplished on no-prep teeth, and the gingitage technique was applied. The gingitage technique was discovered in the past by Ingraham, but its purpose was to open the groove to help in retracting the gingiva and then take the dental impressions [6]. Dental preparation using the BOPT simultaneously involves rotary scraping of the groove, which induces bleeding and creates a space between the inner walls of the groove and the axial walls of the tooth. This space is filled with blood, and a clot is later formed. This blood clot supports the temporary restoration and increases the thickness of the gingival tissue [21]. The healing process involves the reattachment and thickening of the gingival tissue, which will mold and adapt to the emergence of a new profile. This method allows the gums to increase in thickness and adapt to the new shape, which increases their stability in the short and long term [6]. The aim of induced bleeding BOPT is to facilitate the access of immature connective tissue cells and epithelial stem cells and stimulate cell differentiation in this favorable environment, which leads to the formation of new periodontal tissue around the restoration. The newly formed periodontal tissues were histologically normal. The temporary restoration was applied for 4 weeks followed by the final restoration. As for the choice of materials, it was decided to fabricate the veneers from lithium disilicate ceramic. The recent development of ceramic materials, especially injected ceramics reinforced with lithium disilicate crystals, has brought us back to ceramic veneers without preparation. These veneers achieve a thickness similar to feldspar porcelain, up to 0.2 mm, and are more easily achieved clinically and laboratory wise due to their better mechanical properties. These restorations can be made, finished, tested, and installed more safely and have greater strength and fracture resistance than powder/liquid systems due to their lower porosity and higher crystal concentration [23]. Farias-Neto et al. [24] noticed that applying no-prep veneers and ceramic fragments is an excellent rehabilitative option, especially when the dental elements are healthy and can be modified exclusively by adding material, and the patient does not suffer any wear on the teeth. Agustin-Panadero et al. [7] described the rehabilitation of the upper anterior region using no-prep veneers with BOPT cervical margins. They noticed that applying BOPT principles allows gingival remodeling without the need for any preprosthetic surgery. Peris et al. [25] suggested that it is possible to correct gingival asymmetry by performing dental preparation without the finish line by applying the BOPT concepts to correct the asymmetry and obtain a harmoniously integrated restoration with optimal periodontal health. Providing a correct periodontal analysis is first performed, which will contribute to successful soft tissue stabilization. A new protocol suggested by D'Arcangelo et al. [26] to optimize no-prep veneer restorations is presented. Their proposed technique is aimed at identifying optimal margins' positions. The margin is positioned at the point of maximum convexity of teeth, avoiding the overcontouring of traditional no-prep veneers. However, the procedure can be appreciated for the marginal accuracy and the resulting esthetic stability. The case reports show that properly managed no-prep veneers can have biologically healthy and aesthetically pleasant tooth-restoration transitions and emergence profiles.

However, recently introduced compounds such as postbiotics [27], lysates [28], and ozonized gels [29] have been demonstrated to have a significant influence on periodontal health. Therefore, future studies are needed to test these adjunctive treatments in combination with no-preparation veneers applying BOPT to test long-term reliability.

This study has limitations. First, the short time follow-up. Second, the present outcomes cannot be generalized. In addition, it is not possible to establish a cause-effect relationship.

## 4. Conclusion

Based on our findings, after a 12-month follow-up, the gingiva was free of inflammation, recession, and plaque, and there was no increase in probing depth. The patient was satisfied with her new smile, especially because of the absence of preparation, anesthesia, pain, and less time in the dental chair.

## Figures and Tables

**Table 1:** Checklist of the procedures described in the present report.

Procedure	Time point
1. Diagnostic assessment	1^st^ session
2. Orthodontic preparation	2^nd^ session
3. Extraoral and intraoral photographs	3^rd^ session (after 9 months)
4. Intrasulcular mapping	
5. Making the diagnostic wax-up	
6. Making the temporary restoration	4^th^ session
7. Gingitage of the sulcus	
8. Ceramic veneers cementation	5^th^ session (after 4 weeks)
9. Giving the patient oral hygiene instructions	
10. Follow-up sessions	4 sessions (after 3, 6, 9, and 12 months)

## Data Availability

The data that support the findings of this study are available from the corresponding author upon reasonable request.
